# Isolation and characterization of phosphate solubilizing bacteria from the rhizosphere of lentil (Lens culinaris M.) collected from Hagere Mariam district, Central Ethiopia

**DOI:** 10.1371/journal.pone.0308915

**Published:** 2024-11-15

**Authors:** Assefa Shegaw Mengesha, Negash Hailu Legesse

**Affiliations:** 1 Gina-Ager Comprehensive Secondary School, North Shewa Zone Amhara National Regional State, Ethiopia; 2 Department of Plant Science, College of Agriculture and Natural Resource Science, Debre Berhan University, Debre Berhan, Ethiopia; Universiti Putra Malaysia (UPM), MALAYSIA

## Abstract

Phosphorus plays a crucial role in regulating many of the plant’s metabolic activities by enhancing physiological functions and stimulating biological activities such as nodulation, nitrogen fixation, and nutrient uptake in the soil rhizosphere environment. Inoculants of phosphorus solubilizing bacteria serve as an eco-friendly alternative technology that positively influences both soil sustainability and plant growth. The majority of North Shewa highland areas are characterized by low available phosphorus, primarily acidic, and exhibit strong phosphorus absorption. The objective of this study was to isolate and identify phosphorus solubilizing bacteria from the rhizosphere of lentils and characterize their phosphate solubilizing activity. The cultural, biochemical, physiological microbial analysis was conducted in the microbiology laboratory, department of biology. Pikovskaya’s medium was utilized for the isolation, screening, and maintenance of phosphate solubilizing bacteria. Phosphate Solubilizing Bacteria were isolated using tri-calcium phosphate as the sole source of phosphorus in indicator plates. Fifteen phosphate solubilizing bacteria were isolated from lentil rhizosphere soil samples, among which six were the most efficient phosphate solubilizers designated as PSBYE, PSBYR, PSBYM, PSBYL, PSBW, and PSBSW. All isolates notably solubilized tri-calcium phosphate compared to the uninoculated control. The highest phosphorous solubilization was observed from the isolate PSBYL, with a value of 10.61mg/50ml, followed by PSBW with a value of 9.08 mg/50ml. The decrease in pH value correlated with the levels of tri-phosphate solubilization in the PVK broth by the PSB isolates. The pH dropped to 4.64 from the initial pH of 7.2 when grown in the broth, which suggests that the production of organic acids is likely the primary mechanism for phosphate solubilization.

## 1. Introduction

Lentil (*Lens culinaris* Medikus) is a crucial cool-season legume crop in Ethiopia [1–4]. Lentil is cultivated in various cropping systems, including mono-cropping, mixed cropping, intercropping, and relay cropping systems, under both irrigated (10%) and rain-fed conditions (90%) [5] in most lentil growing regions of the world [6]. The lentil is appreciated for its high protein content, which is twice that of cereals [7]. It is an excellent source of easily digestible protein and has fewer anti-nutritional factors compared to other legumes [1]. As a legume, it fixes atmospheric nitrogen through root nodules with the help of rhizobium bacteria, replenishing soil nitrogen supplies and aiding in reducing the application of nitrogenous fertilizers [8]. The nitrogen fixations are particularly affected by phosphorus deficiency, as a low supply of phosphorus inhibits effective nodulation and slows down the biological nitrogen-fixation process [9–11].

Phosphorus is the second most important macronutrient needed for young tissues and vital for plant physiological and biochemical activities. It performs numerous functions related to the growth, development, and metabolism of the plant [12]. It regulates many of the plant’s metabolic activities by enhancing physiological functions. It plays a crucial role in stimulating biological activities like nodulation, nitrogen fixation, and nutrient uptake in the soil [1], leading to higher yields of legume crops in the rhizosphere environment [8]. The application of phosphorus mitigates the adverse effects of drought on physiological parameters and enhances yield under conditions of water stress [13]. Phosphorus, mainly entering the roots as H_2_PO_4_ via the soil solution, influences roots traits such as root length and root hairs of lentils. Lentils suffering from phosphorus deficiency exhibit increased length of primary roots, length and number of lateral roots [1, 14]. The increase in lateral roots is greater than that of the primary root, resulting in an increased root surface area [8]. An increase in root surface area enhances the absorption of phosphorus from soils deficient in phosphorus. Improved morphological (root length and root hairs) and physiological (exudation of phosphatase enzymes) root traits facilitate the efficient utilization of soil nutrients [6]. Phosphorus deficiency increases the proline content in lentil roots, which may function as a regulatory molecule and trigger multiple responses that are components of the adaptation to abiotic stresses [15].

Phosphorus exists in the soil in both mineral and organic forms [16]. A considerable amount of organic phosphorus (20 to 80%) is found to be inactive [16]. Mineral Phosphorus can become inaccessible due to fixation or precipitation reactions with cations such as Ca^2+^ and Mg^2+^ in alkaline soil or Fe^3+^ and Al^3+^ in acidic soil. Although most agricultural soils have substantial phosphorus reserves [17, 18], its availability to plants is restricted due immediate fixation or conversion to an insoluble form, resulting in a low plant-available form [19].

A large portion of soluble inorganic phosphate, when applied to soil as a chemical fertilizer, quickly becomes immobilized and inaccessible to plants shortly after application and losses occur due to leaching in sandy soils or fixation in clay and calcareous soils [20]. The availability of phosphorous in soil to plants is highly dependent on pH and soil type, with maximum P availability occurring at near-neutral pH [21]. In Ethiopia, intensive use of chemical fertilizers is being employed to increase crop productivity. However, these fertilizers are rapidly immobilized and become inaccessible to plants, potentially leading to an overall reduction in soil fertility after application [1, 22].

Microorganisms with the potential to solubilize phosphate enhance the availability of soluble phosphate, thereby promoting plant growth [23, 24]. They support plant growth by fixing nitrogen, solubilizing phosphate, and producing plant growth-promoting regulators [25, 26]. Biofertilizers, in turn, help maintain soil health, minimize environmental pollution, reduce costs, and serve as a substitute for chemical fertilizers [27]. Phosphate-solubilizing bacteria (PSB) play a crucial role in making phosphate ions available to plants, stimulating plant growth by up to 25% [28, 29]. The most effective PSBs are the species of Pseudomonas, Rhizobium, and Bacillus [9, 30]. These bacteria solubilize phosphates through mechanisms such as chelation and the production of extracellular enzymes [9].

Inoculants of PSB can serve as an environmentally friendly alternative technology [25], positively impacting both soil sustainability and plant growth [31], under phosphorus-deficient conditions [9, 10]. The capacity of isolates to solubilize phosphate can be assessed using the plate technique method on Pikovaskaya’s agar, which is based on the clearing zone around the bacterial growth following the incubation period [32].

Most of the highland areas in North Shewa are characterized by low available phosphorus, primarily acidic, and exhibit strong phosphorus absorption [33, 34]. These factors adversely affect the growth of lentils and other pulse crops [1]. The low phosphorous content in the soil has resulted in a decrease in lentil production compared to other leguminous crops [31, 35–37]. The objective of this study was to isolate and identify PSB from the rhizosphere of lentils and soils as well as to characterize their phosphate solubilizing activity (PSA).

## 2. Materials and methods

### 2.1. Study area description

The study was carried out at Debre Berhan University (DBU), situated in Debre Berhan town, 130 km north of the capital city, Addis Ababa. The microbial analysis was performed in the Microbiology Laboratory, department of Biology. The study area is located between latitudes 10.0947 N and longitudes 39.4864 E. Soil and lentil samples were collected from the Hagere Mariam district in the North Shewa Zone, which is 130 km northeast of Addis Ababa and 80 km southeast of Debre Berhan town.

### 2.2. Study design

The study involved isolating Phosphate Solubilizing Bacteria (PSB) from rhizosphere soils of lentils grown in various environmental conditions. The isolates were identified through different morphological and biochemical tests and optimized under different physiological conditions.

### 2.3. Sample collection and size

Soil samples, along with the roots of healthy plants, were randomly collected from five randomly selected agricultural fields. The sampling sites were spaced at intervals of 3 to 5 km, and samples were taken from a depth of 15–20 cm from the rhizosphere soil of lentils in October and November 2019. A total of 20 soil samples were collected, stored in polythene bags, and transported to the Debre Berhan University (DBU) microbiology laboratory. The samples were stored aseptically at 4°C until analysis [38].

### 2.4. Isolation and identification of phosphate-solubilizing bacteria

After conducting cultural characterization, Pikovskaya’s medium employed for the extraction, screening, and preservation of phosphate solubilizing bacteria [39]. Phosphate Solubilizing Bacteria were isolated using tri-calcium phosphate as the sole source of phosphorus in indicator plates [35]. These bacteria have the ability to solubilize insoluble inorganic phosphate compounds, such as tri-calcium phosphate. The isolation process was done using the serial dilution plate count method and Pikovskaya’s medium, a selective medium for isolating Phosphate Solubilizing Bacteria (PSB) [39, 40]. The soil suspension from the rhizosphere was serially diluted and poured onto plates containing Pikovskaya’s Agar. These plates were incubated at a temperature of 30± 2°C for a week. The solubilization of phosphate was indicated by the appearance of a clear zone around the bacterial colonies. Colonies with clear zones were individually isolated onto fresh Pikovskaya’s agar plates and incubated at 30 ± 2° C.

Each soil sample weighing one gram was suspended in 90 ml of phosphate buffer saline (pH 7) in a 250 ml flask and serially diluted. The suspensions were shaken for 30 minutes to break up clumps. Serial dilutions of 10^−1^, 10^−2^, 10^−3^, and 10^−4^ were prepared [41]. From the 10^−3^, and 10^−4^ dilutions, a 0.1 ml of the suspension was transferred onto a Petri-dish containing Pikovskaya’s (PVK) medium. The suspension was evenly spread on the Petri-dish using a glass rod spreader and incubated at 30±2°C for 5 days [42]. Colonies that showed clear zones were selected and purified using the streak plate method [38, 43].

Bacterial colonies were cultivated on Pikovskaya’s medium agar plates to observe the formation of halo zones, which indicate P-solubilization activity. The solubilization index was calculated using the following formula of

$$SI=\frac{colony\;diametre+clearing\;zone}{colony\;diametr}$$

The selected phosphate solubilizing bacterial isolates were identified based on various morphological, physiological, and biochemical characteristics [44]. Standard biochemical tests listed in the Berge’s Manual of Determinative Bacteriology were used for identification purpose [45].

#### 2.4.1. Morphological identification

Bacterial colonies that showed halo zone formations were inoculated on PVK media and cultured at 30°C for 48 hours. Bacteria were identified based on various morphological characteristics such as shape, size, color, and gram staining [9, 46].

#### 2.4.2. Cultural characteristics

The cultural characterization of the isolates was observed by examining various characteristics of the colonies such as shape, size, elevation, surface, margin, color, and pigmentation [47].

#### 2.4.3. Gram staining

The isolated PSB strains were subjected to Gram staining using the standard procedure. The stained cells were observed under a compound microscope to determine their Gram reaction [47].

### 2.5. Biochemical identification

The production of catalase, oxidase, and ammonia, the hydrolysis of starch, motility test, and citrate utilization are valuable criteria for differentiating and identifying various types of Phosphate solubilizing bacteria [38]. Therefore, the positive bacterial isolates were qualitatively analyzed for their citrate utilization, oxidase production, starch hydrolysis, and catalase-producing capabilities [47].

#### 2.5.1. Motility test

The isolates were inoculated by stabbing into a motility agar medium and incubated for 3–5 days at 30±2°C. Diffused growth throughout the test tube indicated motility, while growth only along the stab indicated non-motility [47].

#### 2.5.2. Starch hydrolysis

A sterile starch agar plate was inoculated with 10 μl of broth cultures from the isolates and incubated at 28±2°C for 48 hours. Post incubation, the plates were treated with iodine solution. The appearance of a clear zone around the colony was interpreted as a positive result for the test [48].

#### 2.5.3. Catalase test

This test was conducted to detect the presence of the catalase enzyme in bacterial colonies. Fresh cultures of pure isolates were placed on a glass slide and a drop of H_2_O_2_ (30%) was added. The emergence of gas bubbles indicated the presence of the catalase enzyme [48].

#### 2.5.4. Citrate utilization

The isolates were streaked on Simmon’s citrate agar slants and incubated at 28±2°C for 24 hours. A color change from green to blue indicated a positive reaction for citrate utilization [47].

#### 2.5.5. Oxidase test

The overnight culture of the test isolates were spotted and spread on oxidase test media. Isolates that changed color to pink were identified as oxidase positive [41].

### 2.6. Optimization of conditions

Soil microbes depend on the physicochemical properties of soil physical such as pH, temperature, salt concentration, and other conditions like carbon and nitrogen sources [49] since Environmental factors are crucial for maintaining growth and the production of secondary microbial metabolites. Both physical and nutritional parameters influenced the optimal growth of phosphate solubilizing bacteria [37]. The conditions for maximum growth of phosphate solubilization by selected bacterial isolates were optimized by modifying cultural conditions [35].

#### 2.6.1. Growth at different pH and temperature

The PVK medium was used for incubation at a temperature of 30°C [40]. At the optimal pH (pH = 7.0) of the media was used, and the isolates were incubated at different temperatures (24°C, 28, 32°C, 35°C, 37°C, and 40°C). At the optimal temperature (30°C) and at different pH levels of the media (3, 4, 5, 6, 7, 8, and 9) were set using NaOH or HCl as a buffer in media supplemented with 0.5% tri-calcium phosphate as the sole phosphorus source [49].

#### 2.6.2. Utilization of various carbon and nitrogen sources

The effect of various carbon sources like Glucose, Fructose, Sucrose, Maltose, Starch, Lactose, and Nitrogen sources like Ammonium sulphate, Urea, potassium nitrate, and Ammonium nitrate, were tested in PVK Broth [38]. The isolates were checked for solubilization activity in PVK broth amended with tri-calcium phosphate. Inoculation was carried out using a pure colony of individual bacterial cultures from a PVK Agar plate. Test tubes were incubated at 30°C for 5 days. After incubation, the optimal growths of PSB were quantified by a spectrophotometer,(spectrophotometer reading), the best carbon and Nitrogen sources were determined [49].

#### 2.6.3. Growth at various salt concentrations

Different salt concentrations of NaCl (0, 1, 2, 3, 4, and 5) were added to the Pikovskaya’s broth media and incubated at 30°C and PH = 7.0. The growths of the isolates in the PVK broth were checked after incubation of test tubes at 30°C for 5 days. Then after, the phosphate solubilization by the isolates was quantified using a spectrophotometer, and the presence of growth indicated the salt tolerance level of the microbes [49, 50].

#### 2.6.4. Characterization of phosphate solubilizing bacteria

The characterization of PSB was conducted under *in vitro* conditions by measuring the halo zone, determining the pH change of the medium, estimating the available phosphorous and organic acids [9]. Positive cultures were screened by observing transparent halo zones in Pikovskaya’s medium, which is due to the solubilization of insoluble tri-calcium phosphate into a soluble form. This solubilization is due to the production of organic acids, leading to a lowering of pH in the medium [37, 38].

#### 2.6.5. Measurement of solubilization zone

A qualitative estimation of all the selected phosphate solubilizing bacteria was conducted by growing them on PVK medium after a 7-day incubation period [32]. Colonies of PSB were detected by clear zones of solubilization around them [32, 40]. After the incubation period, the diameters of the solubilization zone produced around the colonies were measured [51]. The efficiency of phosphate solubilization was identified by measuring the total halo zone of the colony and the colony diameter [40]. The zone of phosphate solubilization around the colonies was measured, and the Solubilization index was calculated [52]. The Solubilization index (SI) is calculated as follows:

Solubilizationindex(SI)=(colonydiameter+halozonediameter)/(colonydiameter)

#### 2.6.6. Measurement of available phosphorous in broth media

A quantitative estimation of the amount of inorganic phosphate (Pi) released from phosphate solubilizing bacteria in the broth of PVK medium was conducted [53]. 1ml of each culture was inoculated separately into 50 ml of sterile PVK broth in 100 ml Erlenmeyer flask and incubated at 28 ± 2°C for 7 days, along with uninoculated media serving as a control. After the 3^rd^, 5^th^, and 7^th^ day of incubation at 30° C, cell-free supernatant (CFS) was taken and centrifuged at 10000 rpm for 20 minutes [9, 54–56]. Then, the amount of soluble phosphate was measured by the vanadomolybdate phosphoric yellow color method [57]. The amount of Pi released in the broth by the PSB isolates was estimated at 3, 5, and 7 days after inoculation). The available Pi content in the flask broth was estimated by the phosphor molybdic blue color. For the determination of solubilized P, a standard curve of KH_2_PO_4_ using 2, 4, 6, 8, 10, 12 ppm solutions was prepared [38].

#### 2.6.7. Change in pH of the mediu

Many phosphate solubilizing bacteria release extracellular metabolites into the growth medium (PVK broth), producing organic acids and are responsible for lowering the pH, resulting in the solubilization of phosphorus [11]. The lowering of pH affects the solubilization of phosphates in the medium [54]. The initial pH was measured when the solutions prepared the broth and after the addition of tri-calcium phosphate (TCP) with the bacterial medium. The pH drops significantly during the incubation of solubilization of phosphate [28]. After the incubation period, the pH was measured at different periods of growth, and the initial pH and change in pH were recorded on the 3^rd^, 5^th^, and 7^th^ day by a digital pH meter [51].

#### 2.6.8. Estimation of organic acid production

The production of organic acid by the PSB isolates was determined by measuring the total titrable acidity of the culture filtrate. PSB isolates were inoculated into Pikovaskaya’s broth and allowed to grow for a period of 7 days. After the incubation period, the culture was centrifuged to remove the cell biomass. Two millilitres of the culture filtrate was then taken in a flask, a few drops of phenolphthalein were added, and it was titrated against 0.01 N of sodium hydroxide. The volume of alkali consumed by the culture filtrate represented the total titrable acidity of the culture filtrate, which was expressed in ml of 0.01 N NaOH concentration [58].

#### 2.6.9. Assay for indole acetic acid production

To detect the production of Indole acetic acid, cultures of the isolates that were in their exponential growth phase were incubated in nutrient broth (pH 7.0) supplemented with L-tryptophan 500ml/g and incubated at 28–30˚C for one week. The supernatants of the isolates were collected by centrifugation at 3000 rpm for 30 minutes. Two ml of the supernatant from each isolate was transferred separately into a fresh tube, to which 2–3 drops (100 μl) of O-phosphoric acid and 4 ml of Salkowski reagent (50ml 35% of perchloric acid and 1ml 0.5 FeCl3 solution) were added. The mixture was then incubated in the dark for 30 minutes for the development of a pink color, indicating IAA production [59].

#### 2.6.10. Nitrogen-fixing activity

A single colony grown on nitrogen-free medium was taken and inoculated into G-NFMM containing 0.0025% (w/v) bromothymol blue solution (BTB) [60]. The PSB strains were inoculated on a modified nitrogen-deficient. After one week of incubation, the appearance of a blue-green color confirmed nitrogen-fixing activity [32].

#### 2.6.11. Ammonia production

The production of ammonia was qualitatively detected using the method given by Cappuccino and Sherman. Bacterial isolates were grown in peptone water for 2–3 days at the optimum growth temperature. After incubation, 1ml of Nessler’s reagent was added to each tube. Tubes showing a faint yellow color indicated a small amount of ammonia, and a deep yellow to brownish color indicated a maximum amount of ammonia [60]. The research was not involving humans and animals as experimental units so that the research was conducted by keeping the research ethics.

### 2.7. Statistical analysis

All the collected data were subjected to the analysis of variance (ANOVA). Mean separation was performed using the least significant differences (GLM) procedure at a 5% probability level. Pearson correlation analysis was used to explore the relationships between solubilized P, organic acid activity, and pH values.

## 3. Results and discussion

### 3.1. Isolation of phosphate solubilizing bacteria from lentil rhizosphere

The soil samples were collected and screened for PSB on PVK medium. Phosphate Solubilizing Bacterial (PSB) colonies with high potential phosphate solubilization ability were selected and used to study their impact on the growth of lentil plants in pots. Fifteen phosphate solubilizing bacteria (PSB) were isolated from lentil rhizosphere soil samples, among which six were the most efficient phosphate solubilizes, which had above 50% of SI (solubiliz1ation index) were selected. The selected six efficient phosphate solubilizing bacteria were designated as PSBYE, PSBYR, PSBYM, PSBYL, PSBW, and PSBSW.

All the chosen phosphate solubilizing bacteria were phenotypically distinguished based on their various morphological, biochemical, and physiological traits and grouped under the genera of bacillus. The six selected isolates of PSB exhibited diverse morphological and biochemical characteristics. The PSBYE and PSBYR isolates were larger, while the PSBW and PSBSW isolates were smaller. Most of the selected isolates had a flat elevation, but the PSBYR and PSBYM isolates had a raised elevation. The PSBYE and PSBYR isolates had undulated edges, while the PSBYM and PSBYL isolates had loblet edges. Most of the isolates were white, but some were yellow, and the PSBYR isolate was cream-colored (Table 1). Most of the isolates had an irregular shape, whereas the PSBW and PSBSW isolates were circular. The colony shapes of all isolates were rod-shaped. All six isolates were Gram positive, tested positive for starch hydrolysis, catalase test, citrate utilization, motility test, and ammonia production. Most of the isolates, with the exception of PSBSW, tested negative for the oxidase test (Table 1).

**Table 1:** Morphological and biochemical characteristics of phosphate solubilizing bacteria.

Characteristics	Isolates					
	PSBYE	PSBYR	PSBYM	PSBYL	PSBW	PSBSW
Size	large	large	medium	medium	small	small
Elevation	flat	raised	raised	flat	flat	flat
Edge	undulate	undulate	lobet	lobet	entire	entire
Color	yellow	creamy	yellow	white	white	white
Form	irregular	irregular	irregular	irregular	circular	circular
Gram Rex	+	+	+	+	+	+
Cell shape	rod	rod	rod	rod	rod	rod
Starch Hydrolysis	+	+	+	+	+	+
Citrate Utilization	+	+	+	+	+	+
Catalase test	+	+	+	+	+	+
Oxidase test	-	-	-	-	-	+
Motility test	+	+	+	+	+	+
Ammonia Production	+	+	+	+	+	+
Probable genera	Pseudomonas			Bacillus		

### 3.2. Optimization of physiological conditions

The PSB isolates demonstrated significant variation in growth under different environmental stresses and exhibited varying abilities to withstand adverse environmental conditions. These bacteria grew under different physicochemical parameters such as pH, temperature, salt, carbon, and nitrogen sources, but it is crucial for them to solubilize phosphorus efficiently under optimal growth conditions. It is important to determine whether PSB isolates can survive in conditions of high or low pH, salt, and temperature.

The isolate PSBYM demonstrated the greatest growth, achieving 2.974 at 28°C and 2.835 at 32°C. On the other hand, the isolate PSBSW exhibited the lowest growth of 0.013 at 40°C, followed by the isolate PSBYE with a growth of 0.029 at 24°C. When comparing the average growth of isolates at different temperature ranges, the isolate PSBYM displayed the greatest growth of 1.985, followed by the isolate PSBW with a growth of 1.418 (Table 2). The isolates PSBYR and PSBSW recorded the lowest growths of 1.017 and 1.180, respectively. In terms of the effect of temperature on different isolates, the highest average growth of 2.306 was recorded at 28°C, followed by 1.857 and 1.702 at 32°C and 34°C, respectively. The lowest average growth of 0.388 was recorded at 40°C, followed by 0.733 at 24°C (Table 2). The optimal temperature range for the growth of PSB bacteria was determined to be between 28-32°C, as PSB bacterial strains are mesophilic.

**Table 2:** The effect of different temperature (°C) ranges on the growth of phosphate solubilizing bacterial isolates at Debre Berhan University Microbiology laboratory during 2019.

Isolate	Temperature in °C						
	24	28	32	35	37	40	Mean
PSBYM	1.705a	2.974a	2.835a	1.913a	1.238c	1.247a	1.985a
PSBW	1.489b	2.335c	1.638d	1.579d	1.400b	0.065d	1.418b
PSBSW	0.987c	1.986d	1.531e	1.863b	0.697e	0.013f	1.180e
PSBYR	0.119d	1.980d	1.774c	1.307e	0.882d	0.042e	1.017f
PSBYL	0.067e	2.581b	1.535e	1.634c	1.826a	0.240c	1.314c
PSBYE	0.029f	1.977d	1.827b	1.915a	1.274c	0.722b	1.291d
Mean	0.733	2.306	1.857	1.702	1.220	0.388	1.368
LSD (0.05)	0.014	0.074	0.025	0.015	0.042	0.023	0.021
CV (%)	1.059	1.753	0.741	0.469	1.899	3.249	0.862

CV = coefficient of variation, LSD = least significant difference.

Similar letters along the column means there is no significant difference

**Fig 1:**
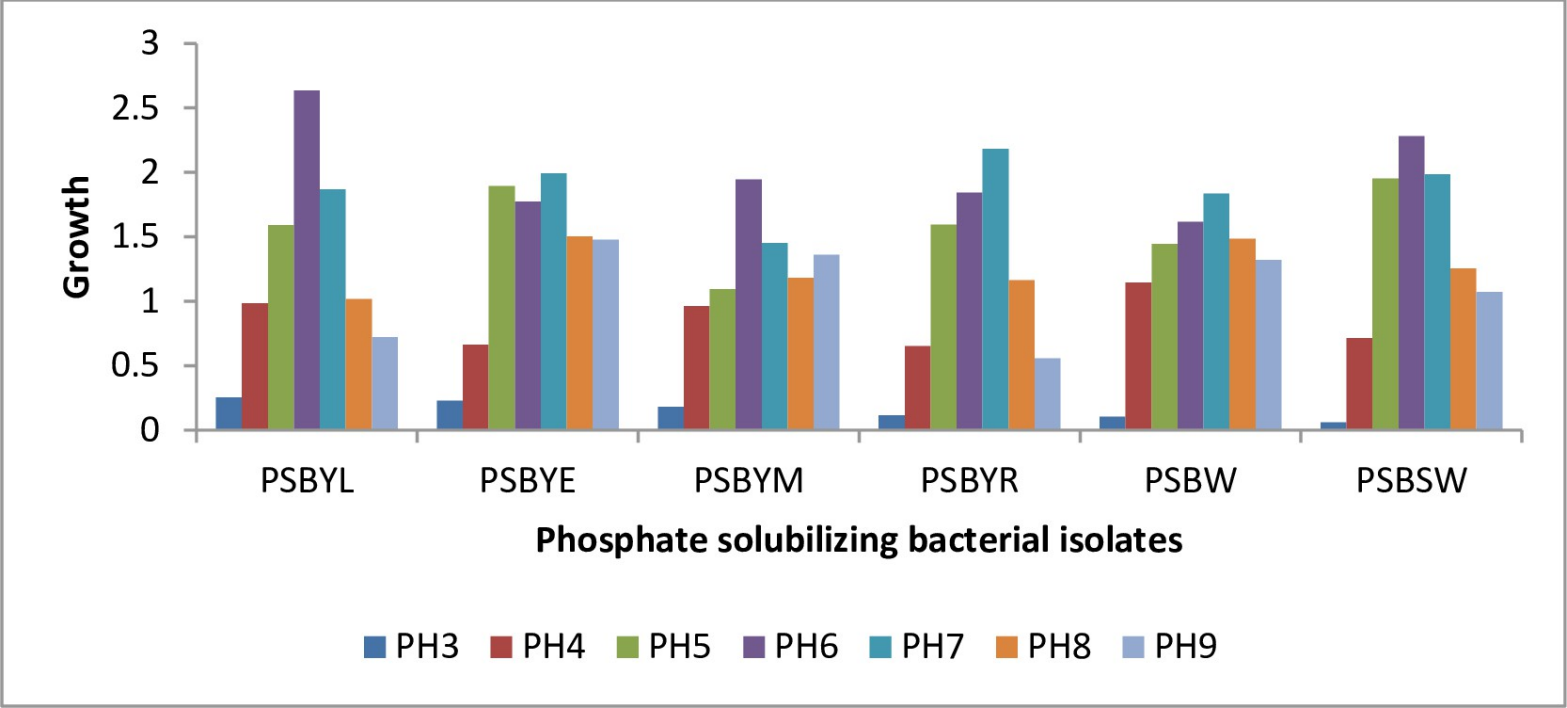
The impact of different pH ranges on the growth of phosphate solubilizing bacterial isolates at Debre Berhan University Microbiology laboratory in 2019.

The isolate PSBYL showed the highest growth, recording a growth rate of 2.638 at a pH value of 6. This was closely followed by the isolate PSBYM, which had a growth rate of 2.282 at the same pH value. Conversely, the isolate PSBSW exhibited the lowest growth of 0.063 at a pH value of 3, followed by the same isolate with a growth rate of 0.075 at a pH value of 9 (Fig 1). When comparing the average growth of the isolates across different pH ranges, the isolate PSBYE displayed the highest growth rate of 1.362, followed by the isolate PSBYL with a growth rate of 1.298 (Table 3). The isolate PSBYR had the lowest growth rate of 1.160, followed by the isolate PSBYM with a growth rate of 1.217 (Fig 1). In terms of the effect of pH on the growth of different isolates, the highest average growth rate of.2.017 was observed at a pH value of 6. This was followed by growth rates of 1.887 and 1.596 at pH values of 7 and 5, respectively. The lowest average growth rate of 0.158 was recorded at a pH value of 3, followed by a growth rate of 0.856 at a pH value of 4. The optimal pH range for the growth of PSB bacteria was determined to be between 6 and 7, as PSB bacterial strains are neutrophilic (Fig 1).

**Table 3:** The effects of varying sodium chloride (NaCl) concentrations on the growth of phosphate solubilizing bacterial isolates, as observed at the Debre Berhan University Microbiology laboratory in 2019.

Isolate	Concentration of Sodium chloride (NaCl) in Percentage						
	0	1	2	3	4	5	Mean
PSBSW	0.728a	1.218c	1.340f	1.225d	1.211c	1.114c	1.139d
PSBYM	0.710b	0.713d	1.425e	1.230d	2.253a	1.459a	1.298c
PSBYL	0.319c	0.126e	1.606d	1.459c	1.230c	1.206b	0.991e
PSBW	0.100d	2.203a	2.058b	1.567b	1.363b	1.113c	1.401a
PSBYE	0.046e	1.220c	2.246a	1.831a	1.302b	1.431a	1.346b
PSBYR	0.046e	1.624b	1.816c	1.140e	1.002d	1.014d	1.107d
Mean	0.325	1.184	1.749	1.409	1.394	1.223	1.214
LSD (0.05)	0.015	0.126	0.040	0.051	0.064	0.029	0.045
CV (%)	2.455	5.865	1.264	1.986	2.533	1.298	2.028

CV = coefficient of variation, LSD = least significant difference.

Similar letters along the column means there is no significant difference

The isolate PSBYM showed the highest growth, with a rate of 2.253, at a Sodium chloride (NaCl) concentration of 4%. This was closely followed by the isolate PSBYE, which had a growth rate of 2.246 at a NaCl concentration of 2%. In contrast, the isolates PSBYR and PSBYE both exhibited the least growth, with a rate of 0.046, at a NaCl concentration of 0%. When comparing the average growth of the isolates at different NaCl concentrations, the isolate PSBW displayed the highest growth rate of 1.401, followed by the isolate PSBYE with a growth rate of 1.346. The isolate PSBYL had the lowest growth rate of 0.991, followed by the isolate PSBYR with a growth rate of 1.107 (Table 3). In terms of the effect of salt concentration on the growth of different isolates, the highest average growth rate of 1.749 was observed at a salt concentration of 2%. This was followed by growth rates of 1.409 and 1.394 at salt concentrations of 3% and 4%, respectively. The lowest average growth rate of 0.325 was recorded at a salt concentration of 0%, followed by a growth rate of 1.184 at a salt concentration of 1%. The optimal range of salt concentration for the growth of PSB bacteria was found to be between 2% and 3% (Table 3).

The isolate PSBYM showed the highest growth on glucose, with a growth rate of 2.128, closely followed by the isolate PSBSW, which had a growth rate of 1.999 also on glucose. In contrast, the isolate PSBYR exhibited the least growth on maltose, with a rate of 0.106, followed by the isolate PSBSW with a growth rate of 0.146 also on maltose. When comparing the average growth of the isolates across different carbon sources, the isolate PSBYE displayed the highest growth rate of 0.713, followed by the isolate PSBSW with a growth rate of 0.680. The isolate PSBYL had the lowest growth rate of 0.495, followed by the isolate PSBW with a growth rate of 0.571 (Table 4). In terms of the effect of carbon sources on the growth of different isolates, the highest average growth rate of 1.842 was observed from glucose. This was followed by a growth rate of 0.437 from starch. The lowest average growth rate of 0.178 was recorded from maltose, followed by a growth rate of 0.395 from fructose. The optimal carbon source for the growth of PSB bacteria was determined to be glucose (Table 4).

**Table 4:** Effects of the utilization of different carbon sources on the growth of phosphate solubilizing bacterial isolates, as observed at the Debre Berhan University Microbiology laboratory in 2019.

Isolate	carbon sources						
	Glucose	Starch	Fructose	Maltose	Sucrose	Lactose	Mean
PSBSW	1.999b	0.259e	0.837a	0.146c	0.488b	0.351c	0.680b
PSBW	1.992b	0.288e	0.276d	0.188b	0.269c	0.411b	0.571e
PSBYE	1.796c	0.609a	0.439b	0.203ab	0.636a	0.596a	0.713a
PSBYR	1.604d	0.394d	0.402c	0.106d	0.488b	0.560a	0.592d
PSBYL	1.535e	0.511c	0.196f	0.207ab	0.246c	0.275d	0.495f
Mean	1.842	0.437	0.395	0.178	0.396	0.421	0.612
LSD (0.05)	0.042	0.037	0.023	0.023	0.031	0.039	0.012
CV (%)	1.254	4.640	3.166	7.123	4.265	5.078	1.093

CV = coefficient of variation, LSD = least significant difference.

Similar letters along the column means there is no significant difference

The isolate PSBYE, when grown on urea, demonstrated the most significant growth, with a rate of 0.617 followed by the same isolate PSBYE, which had a growth rate of 0.546 when grown on ammonium sulphate (Table 5). Conversely, the isolate PSBSW showed the least growth on potassium sulphate, with a rate of 0.055, followed by the isolate PSBYM with a growth rate of 0.056 also on potassium sulphate (Table 5). When comparing the average growth of the isolates across different nitrogen sources, the isolate PSBYE showed the highest growth rate of 0.381, followed by the isolate PSBYM with a growth rate of 0.164 (Table 5). The isolate PSBYL had the lowest growth rate of 0.126, followed by the isolate PSBYR with a growth rate of 0.154 (Table 5). In terms of the effect of nitrogen sources on the growth of different isolates, the highest average growth rate of 0.285 was observed from urea, followed by 0.237 from ammonium chloride. The lowest average growth rate of 0.078 was recorded from potassium sulphate, followed by a growth rate of 0.081 from ammonium sulphate. The optimal nitrogen source for the growth of PSB bacteria was determined to be urea, followed by ammonium chloride as a nitrogen source (Table 5).

**Table 5:** The effects of the utilization of different nitrogen sources on the growth of phosphate solubilizing bacterial isolates, as observed at the Debre Berhan University Microbiology laboratory in 2019.

Isolate	Nitrogen Sources					
	AN	PS	AC	urea	AS	Mean
PSBW	0.233a	0.083b	0.057d	0.201c	0.208b	0.156bc
PSBSW	0.167b	0.055c	0.179c	0.286b	0.103d	0.158bc
PSBYE	0.155b	0.088b	0.499a	0.617a	0.546a	0.381a
PSBYR	0.146bc	0.072bc	0.21bc	0.208c	0.134c	0.154c
PSBYM	0.119cd	0.056c	0.245b	0.291b	0.111d	0.164b
PSBYL	0.098d	0.113a	0.234b	0.105d	0.081e	0.126d
Mean	0.153	0.078	0.237	0.285	0.197	0.190
LSD (0.05)	0.033	0.021	0.047	0.020	0.020	0.009
CV (%)	11.731	14.754	10.761	3.775	5.451	2.702

AN = Ammonium Nitrate, AS = Ammonium Sulphate, PS = Potassium Sulphate, AC = Ammonium Chloride, CV = coefficient of variation, LSD = least significant difference.

Similar letters along the column means there is no significant difference

**Fig 2:**
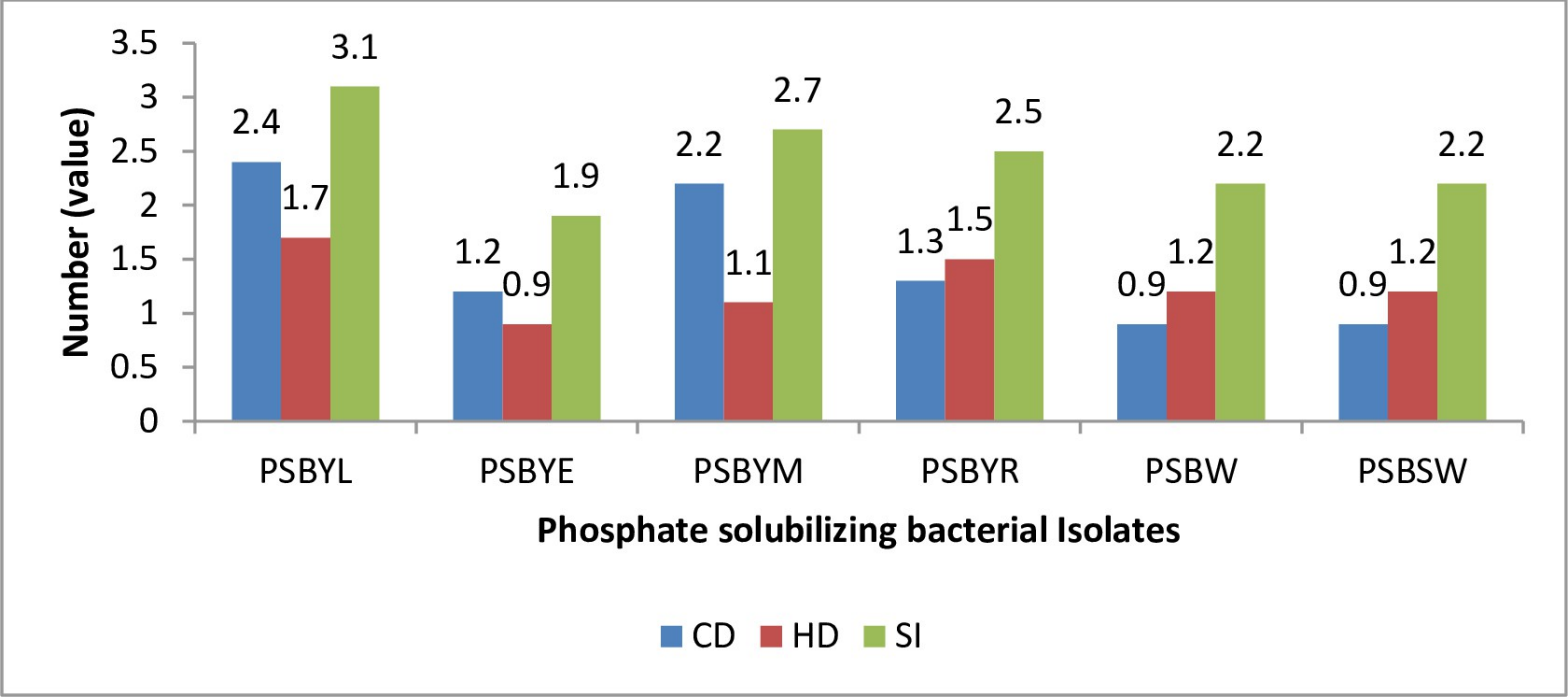
The phosphate solubilization efficiency of PSB isolates, expressed as a phosphate solubilization index (PSI) in a plate assay in Pikovaskaya’s agar, as observed at the Debre Berhan University Microbiology laboratory in 2019, where CD = Colony diameter (cm), HD = Halo diameter (cm), SI = Solubilization index.

The qualitative analysis of the isolates’ ability to solubilize tri-calcium phosphate varied, as reflected in their relative phosphate solubilization index values recorded in Fig 2. The formation of a clear zone indicated a drop in pH during the phosphate solubilization activity in Pikovaskaya’s (PVK) agar plate. The isolate PSBYL exhibited the greatest growth, with a colony diameter of 2.4 cm, followed by the isolate PSBYM, which had a colony diameter of 2.2 cm. The least growth was observed from each isolate of PSBSW and PSBW, with a colony diameter of 0.9 cm, followed by the isolate PSBYE with a colony diameter of 1.2 cm.

Solubilization was evaluated based on the formation of a clear zone. The diameter of the clear zone ranged from 0.9 cm to 1.7 cm. The largest clear zone diameter of 1.7 cm was observed in the isolate PSBYL, followed by PSBYR with a diameter of 1.5 cm. The smallest clear zone of 0.9 cm was observed in the isolate PSBYE followed by PSBYM with a diameter of 1.1 cm. The solubilization zone formed by different isolates ranged from 1.9 to 3.1 after one week of incubation. The highest value of 3.1 was recorded from the isolate PSBYL, followed by PSBYM with a value of 2.7. The lowest solubilization index of 1.9 was recorded from the isolate PSBYE, followed by a value of 2.2 from each isolate PSBW and PSBSW (Fig 2).

For quantitative estimation, the clearing zone activity on an agar plate provided a rough measure of phosphate solubilization potential. The selected phosphate solubilizing bacterial isolates exhibited changes in phosphate solubilization and pH during the solubilization of tri-calcium phosphate in PVK broth over a period of 7 days at an incubation temperature of 28±0.1°C. Therefore, all 6 isolates in this study underwent further quantification in the PVK liquid medium.

In the liquid medium, the solubilization of tri-calcium phosphate by phosphate solubilizing bacteria such as PBYE, PSBYR, PSBYM, PSBYL, PSBW, and PSBSW resulted in a significant decrease in the pH of the culture medium from an initial pH of 7.2. This change was observed on the 3^rd^, 5^th^, and 7^th^ day of the incubation period, with the pH changes recorded at equal intervals.

All isolates notably solubilized tri-calcium phosphate compared to the uninoculated control. The highest phosphorous solubilization was observed from the isolate PSBYL, with a value of 10.61mg/50ml, followed by PSBW with a value of 9.08 mg/50ml (Table 6). The decrease in pH value correlated with the levels of tri-phosphate solubilization in the PVK broth by the PSB isolates. The pH dropped to 4.64 from the initial pH of 7.2 when grown in the broth (Table 6). Therefore, the decline in the pH of the culture supernatants suggests that the production of organic acids is likely the primary mechanism for phosphate solubilization by PSB (Table 6).

**Table 6:** The correlation of pH change, phosphorous solubilization from organic acid (OA) consumed by NaOH.

Isolate	3rd			5th			7th		
	pHC	OAC	Pav.	pHC	OAC	Pav	pHC	OAC	Pav
Control	6.50a	0.001c	0.22f	6.29a	0.005d	0.41d	5.90a	0.008c	0.50g
PSBSW	5.73b	0.011b	3.94e	5.18b	0.018a	5.19c	5.00bc	0.013ab	7.34e
PSBYM	4.96c	0.011b	6.72a	4.75c	0.009c	7.08ab	4.72d	0.013ab	8.12d
PSBYR	4.92c	0.011b	4.96d	4.78c	0.011bc	7.92a	4.74d	0.011b	6.72f
PSBYL	4.92c	0.025a	5.86c	4.74c	0.014b	7.87a	4.93c	0.012b	10.61a
PSBW	4.91c	0.011b	6.27b	4.52d	0.018a	6.66b	5.12b	0.013ab	9.08b
PSBYE	4.83c	0.012b	5.82c	4.68cd	0.009c	5.32c	4.64d	0.015a	8.29c
Mean	5.253	0.012	4.826	4.991	0.012	5.777	5.007	0.012	7.236
LSD (0.05)	0.259	0.003	0.083	0.201	0.003	1.150	0.180	0.002	0.063
CV (%)	2.776	15.916	0.965	2.266	15.853	11.187	2.018	10.285	0.488

OAC = organic acid concentration, Pav = phosphorous available, pHC = pH change

CV = coefficient of variation, LSD = least significant difference.

Similar letters along the column means there is no significant difference

## 4. Discussion

After a week of growth on Pikovskaya’s medium agar plates, halo zones were observed around the colonies of 15 bacterial isolates. The solubilization index, based on the diameter of the colony and the halo zone for each PSB, indicates the efficiency of the conversion of insoluble phosphate into a soluble form, which results in a transparent halo zone around the colony [41]. The six promising isolates were chosen for the greenhouse study based on their significant solubilization of TCP on the PVK media. Since environmental factors are crucial for maintaining growth and the production of secondary microbial metabolites. Both physical and nutritional parameters influenced the optimal growth of phosphate solubilizing bacteria [37]. The conditions for maximum growth of phosphate solubilization by selected bacterial isolates were optimized by modifying cultural conditions [35]. The effective PSB produced indole acetic acid and ammonia, and exhibited nitrogen-fixing activity. Base on cultural morphological and biochemical characteristics, the identified isolates were grouped under the genera Bacillus. The most commonly known PSB belong to the genera Pseudomonas, Bacillus, and Rhizobium [11, 30]. Their optical density (OD) was measured by the spectrophotometer of the broth OD read at 560 nm [43]. Bacterial cells that reached the exponential phase were collected by centrifugation at 1000 rpm for 20 min, washed with distilled water [11, 54].

Based on these morphological and biochemical characteristics, the isolates are supposed to belong to the Bacillus genera. Several studies have identified Bacillus and Pseudomonas as the most dominant phosphorus-solubilizing bacteria, effectively increasing the bioavailability of phosphorus in soil [43, 45]. The optimal temperature range for the growth of PSB bacteria was found to be between 28-32°C, as PSB bacterial strains are mesophilic. This finding aligns with the result of [61], which revealed that the growth of bacteria was greatly influenced by low or high temperatures in the culture medium. The optimal pH range for the growth of PSB bacteria was determined to be between 6 and 7, as PSB bacterial strains are neutrophilic. This study’s findings align with the results of [61], which revealed that the growth of bacteria was greatly influenced by the pH of the culture medium. It was particularly challenging for bacteria to grow in acidic and alkaline conditions, specifically at pH values below 4 and above 9.

The optimal range of salt concentration for the growth of PSB bacteria was determined to be between 2% and 3%.This study’s findings are consistent with the results of [32, 49, 50], which revealed that bacterial growth was greatly influenced by salt concentration. A salt concentration of 5 to 7% was found to inhibit growth and solubilization of phosphorous. The optimal carbon source for the growth of PSB bacteria was determined to be glucose. This is because all PSB isolates preferred to grow on glucose, proving it to be the best carbon source for phosphate solubilization of the isolate in the PKV broth. Starch, maltose, and lactose were moderately utilized as carbon sources.

The pH dropped to 4.64 from the initial pH of 7.2 when grown in the broth. Therefore, the decline in the pH of the culture supernatants suggests that the production of organic acids is likely the primary mechanism for phosphate solubilization by PSB. This study found a significant negative correlation between the release of soluble phosphorous and pH change in the broth [54]. This result confirms that phosphate solubilization by PSB involves the release of metabolites such as organic acids [11]. Most reports indicate that phosphate solubilizing bacteria frequently release gluconic and 2-ketogluconic acid after incubation for 3–7 days, causing a sharp decline in the pH of the broth [58].

## 5. Conclusion

Phosphorus (P) is the second most important macronutrient required for young tissues and is essential for plant physiological and biochemical activities. Phosphorus regulates many of the plant’s metabolic activities by enhancing physiological functions. Phosphorus stimulates biological activities like nodulation, nitrogen fixation, and nutrient uptake in the soil leading to higher yields of legume crops in the rhizosphere environment. Microorganisms with the potential to solubilize phosphate enhance the availability of soluble phosphate, thereby promoting plant growth. Inoculants of PSB can be used as an eco-friendly alternative technology positively influencing both soil sustainability and plant growth, under phosphorus-deficient conditions. The majority of North Shewa highland areas are characterized by low available phosphorus, which are primarily acidic and exhibit strong phosphorus absorption. The aim of this study was to isolate and identify PSB from the rhizosphere of lentils and characterize their phosphate solubilizing activity.

The cultural, biochemical, physiological microbial analysis was performed in the Microbiology Laboratory, Department of Biology. Pikovskaya’s medium was used for the isolation, screening, and maintenance of phosphate solubilizing bacteria. Phosphate Solubilizing Bacteria were isolated using tri-calcium phosphate as the sole source of phosphorus in indicator plates. Fifteen phosphate solubilizing bacteria (PSB) were isolated from lentil rhizosphere soil samples, among which six were the most efficient phosphate solubilizes. The selected six efficient phosphate solubilizing bacteria were designated as PSBYE, PSBYR, PSBYM, PSBYL, PSBW, and PSBSW. All isolates notably solubilized tri-calcium phosphate compared to the uninoculated control. The highest phosphorous solubilization was observed from the isolate PSBYL, with a value of 10.61mg/50ml, followed by PSBW with a value of 9.08 mg/50ml. The decrease in pH value correlated with the levels of tri-phosphate solubilization in the PVK broth by the PSB isolates. This study found a significant negative correlation between the release of soluble phosphorous and pH change in the broth. The pH dropped to 4.64 from the initial pH of 7.2 when grown in the broth. Therefore, the decline in the pH of the culture supernatants suggests that the production of organic acids is likely the primary mechanism for phosphate solubilization by PSB.

## Supporting information

S1 Dataset (XLS)
